# Safe Blood Transfusion Practices among Nurses in a Major Referral Center in Ghana

**DOI:** 10.1155/2021/6739329

**Published:** 2021-03-02

**Authors:** Agnes Asare Bediako, Rasheed Ofosu-Poku, Andrews Adjei Druye

**Affiliations:** ^1^Directorate of Internal Medicine, Komfo Anokye Teaching Hospital, Kumasi, Ghana; ^2^Directorate of Family Medicine, Komfo Anokye Teaching Hospital, Kumasi, Ghana; ^3^Department of Nursing, University of Cape Coast, Cape Coast, Ghana

## Abstract

Errors in transfusion of blood and blood products can lead to preventable morbidity and mortality. Nurses constitute a significant aspect of the transfusion process as they are the last in the chain of getting blood directly to the patient. They must, therefore, be conversant with the current standard of national and international guidelines on blood transfusion and appropriate management of adverse transfusion events. This study assesses the knowledge and practices of blood transfusion safety among nurses at Komfo Anokye Teaching Hospital. A descriptive cross-sectional design was employed, and structured questionnaire (Routine Blood Transfusion Knowledge Questionnaire) was used to collect data from 279 nurses from seven clinical directorates of the hospital. The data were processed with Stata version 14.0. Variables were analyzed using descriptive statistics, and relationships were drawn using inferential statistics. Over 90% of the respondents had a minimum of a diploma in nursing or midwifery, 63% had performed blood transfusion at least 5 times, and 46% had never received any training on blood transfusion. The mean score obtained in all four categories of blood transfusion knowledge assessed was 29, with 54% of the respondents scoring below the mean. The highest overall score on knowledge was 53%. This indicates that nurses had poor knowledge regarding safe blood transfusion practices as stipulated in the clinical guidelines for blood transfusion by Ghana's National Blood Service. There was no statistically significant relationship between training/experience and knowledge of safe blood transfusion practices. Regular and continuous update training and audit are needed to safeguard patient safety during blood transfusion.

## 1. Introduction

An important aspect of routine clinical practice is transfusion of blood and blood products, on account of the life-saving therapeutic benefits they offer [1]. Blood may be used for replacement in case of hemorrhage or anemia; platelets for patients on chemotherapy or to stop postoperative bleeding; plasma for clotting factors in patients with hemophilia; and immunoglobulin for passive immunity for individuals at the risk of certain infections [2]. There is, therefore, a very high need for blood and blood products, and this need may continue to increase as the burden of chronic diseases such as cancer and renal failure continues to rise with increasing life expectancy [3].

The healthcare system must not only ensure the availability of the needed blood products but also must pay close attention to appropriate management of patients during administration. Facilities must therefore have established safe blood transfusion practice guidelines so that the entire process involved in availability and transfusion of blood is well monitored, managed, and coordinated [4].

The process of transfusion consists of five interrelated phases: blood grouping and cross-matching, patient preparation before blood bag collection, blood pack collection, pretransfusion initiation of nursing responsibilities, and posttransfusion nursing care [5]. Four of these phases are relevant to routine nursing practice. The safety and adequate management of transfusion of blood and blood products, therefore, depends largely on the knowledge and skills of nurses.

One of the undesirable yet common associations with transfusion of blood and blood products is adverse transfusion reactions. Transfusion-related reactions are more often than not underestimated as most of the symptoms are nonspecific and may be mistaken for some other condition. Most studies have found that the majority of adverse transfusion reactions is related to preventable human errors [6–8].

The role of nurses is crucial for proper management of transfusion reactions to achieve desirable results, for two main reasons: the process of transfusion is dominated by nursing-related responsibilities, and nurses constitute the last link in the chain of the transfusion process [9]. Nurses must therefore have acceptable knowledge and competencies in transfusion of blood and blood products.

In Ghana, blood transfusion-related problems remain high despite the existence of guidelines for safe blood transfusion practices by the Ghana National Blood Service. A study on adverse transfusion events at Komfo Anokye Teaching Hospital (a tertiary facility which transfuses approximately 17,000 units of blood and blood products annually) reported that adverse transfusion events were as high as 213 per 1000 transfusions. Approxiamtely 5% of transfusions were also stopped on account of mild transfusion events, which could have been managed and transfusions continued [10].

The causes of this high rate of adverse transfusion-related events are currently unknown as no study has been conducted to investigate this phenomenon. However, it is well established internationally that most causes of adverse transfusion reactions (80%) are attributable to human errors [6]. As nurses and midwives constitute a significant stakeholder in the transfusion process, it could be argued that nurse's knowledge and practices regarding blood transfusion may contribute to the safety or otherwise of blood transfusion. However, until now, no study has assessed the knowledge and practices of nurses regarding blood transfusion at Komfo Anokye Teaching Hospital (KATH). Assessing the knowledge and practices of nurses will provide useful cues to understanding if nurses understand and utilize the prescribed blood transfusion safety protocols, as well as their contribution to adverse transfusion reactions at KATH.

## 2. Methodology

### 2.1. Study Design and Setting

This study employed a descriptive cross-sectional design. The study was carried out at KATH, the second largest tertiary hospital in Ghana, which serves as a referral center for about half of the country. The hospital operates under 13 clinical directorates and has a well-structured Transfusion Medicine Unit, which is responsible for blood transfusion services in the hospital and the region as a whole. Approximately 17,000 units of blood and blood products are transfused annually [10].

### 2.2. Study Population, Sampling, and Data Collection

The target population comprised registered nurses and midwives working in the following clinical directorates of the hospital: Internal Medicine, Surgery, Trauma and Orthopaedics, Obstetrics and Gynaecology, Child Health, Oncology, and Accident and Emergency. Registered nurses and midwives who have worked in the hospital for a minimum of 6 months at the clinical area and who have previous experience of at least observing the process of blood transfusion since they were employed in the hospital were included in the study. However, nurses on probation or undertaking their one-year postqualification internship were excluded.

The study adopted quota and convenient sampling methods to select potential participants for the study. To undertake the quota sampling technique, the number of nurses and midwives in each clinical directorate was obtained from the Human Resource Unit of the hospital. The total population of registered nurses and midwives, according to the hospital's 2017 Annual Report, was 1034. Using a confidence level of 95% and an error margin of 5% (0.05), a sample size of 280 was obtained. Using the following formula, a sample size of 280 was obtained.

A total of 308 nurses were however used, taking into consideration 10% attrition rate. Based on the relative number of nurses and midwives in each clinical directorate, a proportion of respondents was calculated from each of the clinical directorates. The breakdown of the sample size from various directorates is as follows: Internal Medicine 54, Accident and Emergency 51, Obstetrics and Gynaecology 84, Trauma and Orthopaedics 23, Surgery 46, Oncology 7, and Child Health 43. The above respective numbers of nurses and midwives were then conveniently sampled from each of the directorates.

Following is the explanation of formula (1) used in this analysis [11]:

(1) $$\begin{array}{c} n=Z^{2}*\frac{\left(p-1\right)}{d^{2}+\left(Z^{2}*p\left(1-p\right)/N\right)}. \end{array}$$

 
*n* = required minimum sample size = 280
 
*N *= population size of the study area = 1,034
 
*Z* = standard deviation
 
*d* = error margin 5% (0.05)
 
*P *= estimated proportion of nurses who will have high knowledge in blood transfusion = 50%

The study adopted the Routine Blood Transfusion Knowledge Questionnaire (RBTKQ) developed by Hijji et al. [12]. The detailed account of the processes used to formulate the RBTKQ ensured its content validity and reliability. The questionnaires were administered personally and conveniently to the respondents at various clinical directorates included in this study: Internal Medicine, Surgery, Obstetrics and Gynaecology; Trauma and Orthopaedics, Oncology, Child Health, and Accident and Emergency Centre (A&E). Registered nurses and midwives at various wards and units approached, and the study was explained to them. Opportunities were given to respondents to ask questions, and clarification was given. Informed consent was obtained after which the questionnaires were administered to those who accepted to participate in the study. Each respondent was allowed only one questionnaire and was encouraged to answer all aspects of it. As a precautionary measure, all questionnaires given out were counted before and after collection. This enhanced respondents' compliance, thereby increasing the return rate.

Out of 308 questionnaires administered (including 10% of attrition), 281 were received, out of which 279 were valid, 90.58% response rate.

### 2.3. Data Analysis and Management

The sociodemographic characteristics and the key variables were used to measure knowledge and practices and were summarized using descriptive statistics. Descriptive analyses were displayed with frequency distribution tables. Fisher's exact test was used to determine the relationship between sociodemographic attributes and other variables. Software Stata version 14.0 was used to analyze the data.

For all questions (both multiple-choice and single choice answers), the scores for each of the individual options chosen (as the correct answer) by respondents were displayed to provide a clear picture of the knowledge and practice of participants. The correct answers have been written in italics. For each of the phases of the transfusion process, the mean score reflecting only correct responses was also calculated.

### 2.4. Generalizability

Since almost all the clinical directorates of KATH were used, the study is generalizable at KATH; however, it cannot be generalizable in Ghana.

### 2.5. Ethics Statement

Ethical clearance was first obtained from the research subcommittee of the Ghana College of Nurses and Midwives (GCNM). Written permission was then sought from the Director of Nursing of KATH after which the study was registered with the Research and Development Unit of Komfo Anokye Teaching Hospital, and ethical clearance was obtained from the Committee on Human Research Publication and Ethics of Kwame Nkrumah University of Science and Technology. Informed consent of the participants was sought, and voluntary participation, confidentiality, and anonymity were assured.

## 3. Results

The results of the study have been illustrated in Tables 1–11.

From Table 1, most (83.6%) of the respondents were aged between 26 and 35 years, and 77.74% were females. With respect to their level of education, 38.11% held a degree in nursing or midwifery and 58.87% diploma in nursing or midwifery. Respondents had a mean of 5.22 years of experience working in a unit where blood transfusion takes place; 63.49% had performed blood transfusion at least 5 times, and 46.07% had never participated in any kind of formal training or formal continuous professional development session on blood transfusion.

In Table 2, a majority of the respondents, 220 (78.85%), indicated that checking the availability and patency of an intravenous access must take place after bringing blood to the ward, and 228 (83.21%) indicated blood should be collected from the blood bank before premedication is administered, whereas 6 (2.15%) stated that they will refuse to collect and administer blood if the physician's order for transfusion reads, “give one unit of packed cells IV,” 210 (75.27%) stated that they will collect and transfuse blood if the above order is written. A majority of the participants (240 corresponding to 86.02%) would, before carrying out a blood transfusion, inform the patient about the reasons for transfusion, and 68–100 of them (24.37–35.84%) would discuss the risks of transfusion and symptoms of the transfusion reaction as well. Of the respondents, 233 (95.10%) would rightly check baseline vital signs within 30 minutes prior to initiating a blood transfusion. Registered nurses in this study had a mean score of 31.91 for patient preparation before blood transfusion (Table 3).

In Table 4, 169 respondents (62.13%) knew that, after blood pack collection, ensuring patient's identification details are the same as those on the blood bag and the transfusion request form is the first thing to do; 96 (35.42%) respondents knew the most appropriate approach to handling blood immediately after obtaining the blood bag is to be initiating transfusion immediately. Regarding blood warming prior to administration, 90–128 respondents (32.26%–45.88%) knew that the right indications were in exchange blood transfusion in infants, rapid transfusion, and patients with cold agglutinins. 34 respondents (18.99%) knew that the suitable filter size for a blood transfusion set is 170–200 microns. Prior to initiation of transfusion, 252 (93.33%) respondents would ensure that patient's identification details match those on the blood bag, but only 34–44% of participants would ask the patient to state his/her name and date of birth when possible as an essential step in ensuring the right blood is transfused to the right patient. Participants had a mean score of 26.54 for knowledge of pretransfusion nursing activities (Table 5).

From the commencement to the end of blood transfusion, key nursing activities include setting up the rate of flow of the blood or blood product, documentation of relevant information such as vital signs, and observation of the patient for the transfusion reaction. From Table 6, of the 279 respondents in this study, 137 (49.10%), 251 (89.96%), and 247 (88.53%), respectively, knew the aforementioned nursing activities. Of the respondents, 94 (42.53%) knew that the correct rate for starting transfusion of one unit of blood in an adult patient was 120 ml/hour or less. A majority, 187 (69.78%), knew that the maximum duration within which the administration of a unit of blood must be completed to be 4 hours. However, 47 (17.47%) would correctly observe the patient for a possible transfusion reaction within the first 15 minutes of transfusion. Participants had a mean score of 17.88 for knowledge of posttransfusion nursing activities (Table 7).

In order to minimize the risk of the transfusion reaction, the nurse must administer compatible blood, within 4 hours after collection from the blood bank, not transfuse incompatible solutions with blood, and stop transfusion immediately when signs and symptoms of the reaction are observed. From Table 8, only one respondent (0.36) had all the four answers right. 120–210 respondents (43.01–75.27%) knew that tachycardia, chest pain, hypotension, and nausea and vomiting were signs and symptoms of an acute haemolytic transfusion reaction. However, only 21 (7.53%) selected all four appropriate answers together. 80 (30.65%) knew patient identification error was the most common cause of most fatal transfusion reactions. Immediately upon observing manifestations of acute haemolytic transfusion, 239 participants (85.66%) knew that stopping the transfusion was the first step to take, and 21 (7.53%) would also check the vital signs. However, upon observing a mild allergic reaction, 6 (2.16%) knew that the most appropriate immediate action was to slow down the transfusion rate and notify the doctor. Participants had a mean score of 42.00 for knowledge of transfusion complications (Table 9).

In Table 10, 137 respondents (51.12%) said there was no blood transfusion policy on the ward, whereas 54 (20.15%) were not sure if there was one or not. 77 (28.73%) respondents indicated they were aware of the availability of the blood transfusion policy on their ward. However, out of these 77 respondents, 71 (92.21%) had read it, but 6 (7.799%) had never read it.

From Table 11, 54% of the respondents scored less than the mean score of 29%. The highest score obtained was 53%.

Using Fisher's exact test to determine the association between previous training/experience and knowledge on all phases of the blood transfusion process, a statistically significant relationship (*p* value: 0.04) was observed only with patient identification prior to initiating blood transfusion.

## 4. Discussion

### 4.1. Demographic Characteristics

Participants held a minimum qualification of diploma in nursing or midwifery and as such were expected to have the technical know-how relating to the entire blood transfusion process, especially in all issues relating to the practice of nursing or midwifery. Approximately half of the respondents (46.07%) had never participated in any kind of training or professional development session on blood transfusion. Similarly, in a study in Burkina Faso, a far lower proportion of respondents (13.5%) had ever participated in any continuous professional improvement learning activity on blood transfusion. This observation was linked to limited resource availability in Africa leading to less frequent or fewer educational opportunities [13].

### 4.2. Patient Preparation before Blood Transfusion

This study found that 79% of registered nurses would obtain the blood bag from the blood bank before checking the patency of the intravenous access and administering prescribed premedication (Table 2). This approach leads to unusual delay in starting blood transfusion after the blood bag is obtained from the blood bank and may lead to hemolysis and bacterial contamination [14, 15]. This has therefore been discouraged in various blood transfusion guidelines [15, 16]. This finding sharply contrasts with a study among Jordanian nurses in which a majority of them would obtain the blood bag from the blood bank only after ensuring the intravenous line is patent and premedication has been given [12].

Another important aspect of preparing a patient for blood transfusion is obtaining informed consent from the patient/family. This is done by explaining to them the reasons why blood transfusion becomes necessary, its benefits, associated risks, and symptoms of transfusion reactions to watch out for [16]. In this study, 24%–35% of the participants knew the right issues to discuss whilst obtaining informed consent for blood transfusion (Table 2). A previous observational prospective study similarly found that 72% of the participants did not obtain informed consent from the patients and family before initiating blood transfusion [17]. Another observational study of two tertiary hospitals in Nepal also found that 2.4%–8.2% of 86 observations of blood transfusions done involved informing patients and family of reasons for transfusion, benefits, and potential risks whilst seeking informed consent to transfuse [18].

In order to appropriately address the needs of patients, it is essential that doctors give clear instructions or medical orders. In the event of an ambiguous order, the nurse must not collect and administer the blood or blood product, but must seek clarification. In this study, 75% of the participants indicated that they would act on an incomplete medical order to give “packed cells IV” (Table 2). This is similar to, although much higher than, the findings from a study by Khetan et al. in which 87% of participants would act on an incomplete medical order [17].

The mean score of registered nurses and midwives in this study with respect to preparation of patients prior to blood transfusion was 32 (Table 3). Thus, study participants had inadequate knowledge about issues to discuss whilst obtaining informed consent, when to administer premedication, and about ensuring intravenous access prior to transfusion. This finding contrasts with the study conducted in Egypt by Elhy and Kasemy in which a majority of nurses participating in the study had adequate knowledge about appropriate preparation of the patient prior to transfusion [19]. It also contrasts with a study conducted in Cape Coast Teaching Hospital in Ghana by Tetteh , in which nurses were found to have excellent knowledge about patient preparation before transfusion [5].

### 4.3. Knowledge of Pretransfusion Nursing Activities

The most common cause of major adverse transfusion reactions is patient identification errors [6, 20]. It is for this reason that guidelines on transfusion not only discuss this subject but also have detailed procedures to correct patient identification [21, 22]. In this study, although 90% of nurses indicated they would cross-check patient's identification details with those on the blood bag, 62% felt patient identification is the most important pretransfusion nursing activity (Table 4). These results are similar to a Jordanian study in which 70% of study participants knew they had to cross-check patient's identification details with those on the blood bag [12]. In another study in a tertiary hospital in North India, a relatively higher number of participants (98%) knew they had to cross-check patient's details [17].

With respect to identification of patients, it is important for nurses to enquire from all conscious patients their name and date of birth or age and to cross-check with the folder, blood bag, and request form [16]. Failure to adhere to this guideline may lead to patient identification and serious blood transfusion errors. In this study, in spite of the fact that over 90% of nurses knew patient identification was very significant in the transfusion process, only 33%–44% stated that asking the patient to state the name and date of birth was an appropriate practice in identification of the patient. This finding is similar to the study in North India in which only 40% knew the correct procedure [17]. Similarly, in a previous study in France assessing factors associated with poor transfusion knowledge and practice, 12%–36% carried out bedside compatibility [23].

One of the most common practices of transfusion which is not indicated in most cases is blood warming. Blood warming is indicated in cases of rapid/massive transfusion, exchange transfusion in infants, in patients with cold agglutinins, and in trauma situations where core rewarming measures are needed [24]. In this study, 36–46% of registered nurses/midwives knew these right situations in which blood warming is needed (Table 4). Thus, a majority of them (54–64%) would therefore warm blood in inappropriate circumstances, with 16% warming blood in all situations. This finding is similar to the study conducted by Khetan et al. in which 95% of participants in the study would warm blood in the wrong circumstances [17].

Furthermore, 35% of study participants would start transfusion immediately after obtaining the blood bag from the blood bank without having to wrap or warm it (Table 4), whereas in the Jordanian study, 92% of nurses would wrap, warm, or microwave the blood bag before administration to the patient [12]. Only 19% of respondents knew the most appropriate filter size of intravenous tubing for blood transfusion (Table 4). This result is similar to the results from the Jordanian study in which over 70% of nurses either stated the incorrect filter size or stated straightforward that they did not know the required filter size for the blood transfusion intravenous tubing [12].

Another significant pretransfusion nursing activity is the checking and recording of vital signs within 30 minutes prior to initiation of blood transfusion. Of the participants, 95% knew this as the standard practice (Table 4). This sharply contrasts with findings from an observational study in Nepal in which only 2–4% carried out assessment of vital signs within the first 15 minutes of initiating blood transfusion. The difference may be on account of the methodological differences between the studies as knowledge has been shown not to be translated into practice on many occasions [18].

The mean score of participants with respect to pretransfusion nursing activities was 27 (Table 5). Thus, the level of knowledge of pretransfusion nursing activities in this study was poor. Similar findings were reported in a previous study in Aquitaine, France, in which 34.5% of over 1000 nurses had adequate knowledge of activities to be undertaken in the pretransfusion phase [23].

### 4.4. Knowledge of Posttransfusion Nursing Activities

After initiating blood transfusion, nursing responsibilities involve setting the appropriate flow rate of transfusion, documenting all relevant information including assessment findings, and ongoing observation for transfusion reactions [16]. In this study, 40% of the respondents knew the correct rate of transfusion in a normal adult, whereas those who knew that blood must be administered slowly in patients with cardiovascular disease and anemia were 26% and 5%, respectively. Furthermore, only 26–68% of nurses in this study knew the appropriate monitoring to undertake after initiating blood transfusion (Table 6). These results reveal that most nurses in this study had poor knowledge on nursing activities undertaken following the initiation of transfusion. A quasi-experimental study among 55 nurses in a hospital in Egypt similarly found that prior to carrying out the intervention protocol, participants had poor knowledge of nursing care after initiation of transfusion [25].

In this study, 90% of nurses knew they had to document care rendered during the entire transfusion process (Table 6). Although this number looks encouraging, many studies have reported the poor state of nursing documentation and lack of standard within Ghana's Health Service [26, 27]. Kaur et al., in their study assessing the impact of training on knowledge of blood transfusion among clinicians, found that 16% carried out the appropriate documentation prior to the training [28].

The mean score of registered nurses and midwives with respect to knowledge with regard to nursing responsibilities after initiation of blood transfusion, such as routine activities performed after starting the blood transfusion, maximum duration for completing a unit of blood, maximum duration of using a blood administration set for continuous multiple transfusions, and agents compatible with blood, was 18 (Table 7). This result was in agreement with a study conducted by Khalil et al. which revealed that nurses had insufficient level of knowledge about blood transfusion [25]. Similarly, a more recent study conducted in Malaysia by Lee et al. also found that about one-third of participants did not recognize the time for measuring vital signs after initiating blood transfusion, and 80% did not know the possible consequences of transfusing blood over a longer duration than that which is recommended [29].

### 4.5. Practices of Nurses in Monitoring Blood Transfusion and Related Complications

This study found that more than half of respondents (58%) had poor knowledge regarding complications related to blood transfusion (Table 8). This finding is in line with a study conducted by Silva et al. in which about 70% of the study participants had inadequate knowledge about appropriate nursing care in the event of a transfusion complication [30]. Yaghoobi et al. , in their study, assessing transfusion-related knowledge of staff in Iran, however, found that a majority of their participants had adequate knowledge of appropriate transfusion monitoring and required action in the event of a transfusion reaction [31]. The difference between the above studies may be due to institutionally organized periodic refresher courses for nurses in the latter study.

The mean score of participants in the management of transfusion monitoring and complications was 42 (Table 9). Although the mean score in this phase of transfusion is relatively higher than the first three phases discussed above, it is still quite lower than expected. This phase may have scored higher because nurses may be more focused on events related to transfusion-related complications and reaction as compared to the other phases.

### 4.6. Availability and Content of the Transfusion Policy

This study revealed that 71.27% of participants had no knowledge of the availability and content of the blood transfusion policy (Table 10). This result contrasts with the study conducted by Erel et al. in which less than 15% of the participants had no knowledge about the existence of the blood transfusion policy [32]. Furthermore, a prior study conducted by Hijji et al. also found that only 16% of the nurses were unaware of the existing blood transfusion policy [12].

### 4.7. Comparative Analysis of Transfusion-Related Knowledge of Nurses and Their Previous Experience and Training

Using Fisher's exact test, there was no statistically significant relationship between almost all the components assessing adequate knowledge of safe blood transfusion and the previous training or experience of respondents with respect to blood transfusion. This finding corroborates a study in Brazil in which no association was similarly found between knowledge of all phases of the blood transfusion process. The authors found that lower knowledge was associated with learning through experience and from colleagues, as well as inadequate periodic refresher courses or in-service training on blood transfusion [33]. However, findings of this study contrast with a previous study conducted by Mwamwenda in which higher knowledge scores were associated with higher years of experience [34].

### 4.8. Conclusion

Respondents had experience of working in a unit where blood transfusion is done for an average of 5 years. Over 90% of the 279 respondents had a Diploma or Bachelor of Science degree in Nursing or Midwifery, 46% had never participated in any kind of training or professional development session on blood transfusion, and 63% had performed blood transfusion at least 5 times.

This study found that the majority of nurses and midwives had poor knowledge relating to all four phases of the blood transfusion process in which nurses played an instrumental role. They also had gross knowledge deficit about the clinical transfusion guidelines developed by the National Blood Service, Ghana. No statistically significant relationship was found between the previous experience/knowledge of transfusion and the knowledge scores.

Educational curricula in the nursing schools should be upgraded to include transfusion medicine to equip nurses. When these actions, among others, are implemented, there is the possibility of improving the safety of blood transfusion practice, which may translate into the overall subsequent reduction in adverse transfusion reactions in the hospital.

The hospital must provide opportunities for continuous in-service training in transfusion medicine for its clinical staff. The Blood Transfusion Unit needs to develop simplified standard operating procedures based on the clinical blood transfusion policy to be posted on clinical areas where blood transfusion is carried out. Individual nurses must take responsibility in training themselves in blood transfusion medicine.

## Figures and Tables

**Table 1:** Demographic characteristics.

Variable	Category	Frequency	Percentage (%)
Age	21–25	19	7.79
	26–30	109	44.67
	31–35	95	38.93
	35+	21	8.61

Gender	Female	213	77.74
	Male	61	22.26

Educational qualification	Degree in nursing/midwifery	101	38.11
	Diploma in nursing	105	39.62
	Diploma in midwifery	51	19.25
	Masters	8	3.02

The number of times blood transfusion was performed	1–4 times	90	32.37
	5–8 times	51	18.35
	9–12 times	30	10.79
	More than 12 times	93	33.45
	None	14	5.04

Formal training in blood transfusion	Never	123	46.07
	Yes	144	53.93

**Table 2:** Patient preparation before blood transfusion.

Variable	Frequency	Percentage (%)
A nurse should check the availability and patency of an intravenous access line after bringing blood to the ward		
True	220	78.85
*False*	59	21.15

Blood collection from the blood bank should take place before the administration of any prescribed premedication(s)		
True	228	83.21
*False*	46	16.97

What should be the nurse's immediate decision on physician's order of “give one unit of packed cells IV”? (one answer requested)		
Collect blood and clarify the order with the physician prior to administration	53	19.00
Seeking assistance from the head nurse/nursing supervisor	10	3.58
Collect blood and transfuse the patient	210	75.27
*Refuse to collect and administer blood*	*6*	*2.15*

On what issues should the patient be informed before each blood transfusion episode?		
*Reasons for blood transfusion*	240	86.02
Management of the acute transfusion reaction	10	3.58
*Risks of blood transfusion*	68	24.37
*Reaction symptoms*	100	35.84
Possible consequences of rejecting to have the transfusion	4	1.43

When should the baseline vital signs be recorded before initiating blood transfusion?		
Within 2 hours	2	0.82
Within 1 and 1/2 hours	4	1.63
Within 1 hour	6	2.45
*Within 30 minutes*	233	95.10

**Table 3:** Nurses' knowledge on patient preparation before blood transfusion.

Question	Correct	
	Frequency	Percentage (%)
A nurse should check the availability and patency of an intravenous access line after bringing blood to the ward	59	21.15
Blood collection from the blood bank should take place before the administration of any prescribed premedication(s)	46	16.79
What should be the nurse's immediate decision on physician's order of “give one unit of packed cells IV”?	6	2.15
On what issues should the patient be informed before each blood transfusion episode?	68	24.37
When should the baseline vital signs be recorded before initiating the blood transfusion?	233	95.10
Mean score		31.91

**Table 4:** Pretransfusion initiation nursing activities.

Variable	Frequency	Percentage (%)
What is the most important nursing action that the nurse must do with regard to the patient after obtaining the blood pack but before starting the transfusion?		
Check the doctors' order with another nurse	16	5.88
Document baseline vital signs	79	29.04
*Identify the right patient*	169	62.13
Provide information to the patient (or family)	4	1.47
Report high temperature to the doctor	4	1.47

In the ward after obtaining a blood bag, how would you handle blood?		
Wrap the unit with a blanket or bed sheet	24	8.86
Allow blood to wait in room temperature	149	54.98
Immerse the unit in hot water	1	0.37
*Start the transfusion immediately*	96	35.42
Warm in a microwave	1	0.37

When is blood warming prior to administration clinically indicated?		
Each time a unit of blood is to be transfused		
Yes	45	16.13
No	234	83.87
*In exchange transfusion in infants*		
*Yes*	128	45.88
*No*	151	54.12
*In rapid transfusion*		
*Yes*	90	32.26
*No*	189	67.74
*In patients with cold agglutinins*		
*Yes*	110	35.84
*No*	169	64.16
In patients with hypothermia		
Yes	186	66.67
No	93	26.33
What is the suitable filter size of the blood transfusion set?		
90–120	74	41.34
130–160	63	35.20
170–200	34	18.99
210–250	8	4.47

What are the 3 most important steps that a nurse has to follow in order to properly identify the right patient prior to initiating the transfusion?		
*Ask the patient to state his/her name when possible*		
*Yes*	124	44.44
*No*	155	55.56
Call the patient name when possible		
Yes	156	55.91
No	123	44.08
Check the room and bed number		
Yes	39	13.98
No	240	86.02
*Ensure that patient identification details match on the blood bag, ID band*, *and request form*		
*Yes*	252	90.33
*No*	27	9.68
*Ask the patient to state his/her date of birth when possible*		
*Yes*	96	34.41
*No*	183	65.59
Compare the ID band with the blood bag		
Yes	5	1.79
No	274	98.21

**Table 5:** Nurses' knowledge of pretransfusion nursing activities.

Question	Correct	
	Frequency	Percentage (%)
What is the most important nursing action that the nurse must do with regard to the patient after obtaining the blood pack but before starting the transfusion?	150	60.98
When is blood warming prior to administration clinically indicated?	10	3.58
A unit of blood was delivered to the ward at 4.00 PM. What is the best time by which the transfusion should start?	132	47.31
In the ward after obtaining a blood bag, how would you handle blood?	96	34.41
What are the 3 most important steps that a nurse has to follow in order to properly identify the right patient prior to initiating the transfusion?	2	0.72
What is the suitable filter size of the blood transfusion set?	34	12.19
Mean score		26.54

**Table 6:** Posttransfusion initiation nursing activities.

Variable	Frequency	Percentage (%)
What are 3 routine nursing activities a nurse has to perform just after starting the blood transfusion until it ends?		
*Setting up the flow rate*		
*Yes*	137	49.10
*No*	142	50.90
*Documentation of relevant information including vital signs*		
*Yes*	251	89.96
*No*	28	10.04
Flush line using normal saline		
Yes	37	13.26
No	242	86.74
Observation for the transfusion reaction		
Yes	247	88.53
No	32	11.47
Check the patient's identity		
Yes	29	10.39
No	250	89.61

The doctor has prescribed a unit of blood to an adult patient. At what rate would you start this transfusion?		
Not more than 60 mL/hour	92	41.63
*Not more than 120 mL/hour*	94	42.53
Not more than 150 mL/hour	27	12.22
Not more than 200 mL/hour	8	3.62

A unit of blood intended for an adult patient was removed from the blood bank at 4.00 PM. What is the maximum duration when the unit should be totally consumed by the patient?		
2 hours	37	13.81
3 hours	26	9.70
4 hours	187	69.78
5 hours	18	6.79

When and for how long is it essential to physically observe the patient for possible transfusion reaction? (only one answer requested)		
*For the first 10–15 minutes*	47	17.47
*For the first hour*	14	5.20
*Throughout the shift*	35	13.01
*Throughout the transfusion*	173	64.31

**Table 7:** Nurses' knowledge of posttransfusion nursing activities.

Question	Correct	
	Frequency	Percentage (%)
What are 3 routine nursing activities a nurse has to perform just after starting the blood transfusion until it ends?	10	3.70
What may happen to a patient if rapid administration of cold blood is performed through a central venous route terminating in or near the right atrium?	116	41.88
The doctor has prescribed a unit of blood to an adult patient. At what rate would you start this transfusion?	94	33.69
What is the maximum duration each blood administration set could be used for continuous multiple blood transfusions?	5	1.79
In order to initiate a blood transfusion slowly on a 4-month-old infant, at what rate would you start this transfusion during the first 15 minutes?	29	10.39
A unit of blood intended for an adult patient was removed from the blood bank at 4.00 PM. What is the maximum duration when the unit should be totally consumed by the patient?	187	67.03
Slow blood transfusion should be considered for which of the following patients?	12	4.30
Specify which of the following solutions/agents could be safely mixed with transfusion of blood.	3	1.08
A unit of blood was initiated at 2.00 PM and is expected to be completed at 5.00 PM. When should the patient's vital signs be recorded after initiation until completion?	3	1.08
When and for how long it is essential to physically observe the patient for possible transfusion reaction?	47	16.85
Mean score		17.88

**Table 8:** Complications related to blood transfusion.

Variable	Frequency	Percentage (%)
What interventions could minimize the risk of the patient experiencing acute transfusion reaction? (four answers required)		
*Administration of blood that is compatible with that of the recipient*		
*Yes*	251	89.96
*No*	28	10.04
Starting the transfusion within 20 minutes after collection from the blood bank		
Yes	46	16.49
No	233	83.51
*Administering a unit of blood to the patient within 4 hours after collection*		
*Yes*	133	47.67
*No*	146	52.32
Taking the history from the patient		
Yes	21	7.53
No	258	92.47
*Not transfusing drugs or solutions that are incompatible with blood*		
*Yes*	131	46.95
*No*	148	53.05
*Stopping blood if there are signs and symptoms of the transfusion reaction*		
*Yes*	58	20.79
*No*	221	79.21
*Correct score*	1	0.36
*Wrong score*	278	99.64

What signs and symptoms indicate that the patient is developing an acute haemolytic transfusion reaction?		
Tachycardia		
Yes	210	75.27
No	69	24.73
Productive cough		
Yes	30	10.75
No	249	89.25
Chest pain		
Yes	167	59.86
No	112	40.14
Bradycardia		
Yes	71	25.45
No	208	74.55
Hypotension		
Yes	153	54.84
No	126	45.16
Shallow respiration		
Yes	137	49.10
No	142	50.90
Nausea/vomiting		
Yes	120	43.01
No	159	56.99
Neck pain		
Yes	30	10.75
No	249	89.25
Correct score	21	7.53
Wrong score	258	92.47

What should be done immediately when signs and symptoms of the acute haemolytic transfusion reaction are seen?		
Stop blood transfusion		
Yes	239	85.66
No	40	14.34
Inform the nursing supervisor		
Yes	1	0.36
No	278	99.64
Check the patient's vital signs		
Yes	21	7.53
No	258	92.47
Notify the doctor and begin emergency treatment according to the medical order		
Yes	8	2.87
No	271	97.13

What is the commonest cause of the most fatal transfusion reactions?		
Warming blood to more than 37°C	9	3.45
Error in blood bank testing	94	36.02
Antibodies in the Rh system	78	29.02
Identification error of the patient	80	30.65

What is the first action that the nurse should take to handle a patient with mild allergic transfusion reaction?		
Stop the transfusion and notify the doctor	239	88.85
Notify the doctor and slow the transfusion rate	2	0.74
Slow the transfusion rate and notify the doctor	6	2.23
Check the patient's vital signs	21	7.81
Notify the senior nurse	2	0.37

**Table 9:** Knowledge of nurses on blood transfusion complications.

Question	Correct	
	Frequency	Percentage (%)
What interventions could minimize the risk of the patient experiencing acute transfusion reaction?	11	3.94
What signs and symptoms indicate that the patient is developing an acute haemolytic transfusion reaction?	21	7.53
What should be done immediately when signs and symptoms of the acute haemolytic transfusion reaction are seen?	47	16.85
Due to an emergency, a unit of blood collected at 8.00 PM was kept in the nurses' station until 9:30 PM. What should the nurse do with blood?	179	65.09
A patient has sustained a mild allergic transfusion reaction. What is the usual presenting complaint?	231	83.09
What is the first action that the nurse should take to handle a patient with mild allergic transfusion reaction?	6	2.16
What is the commonest cause of the most fatal transfusion reactions?	80	28.78
Before administering blood, when is it acceptable not to check patients' details at the bedside?	177	63.44
Mean score		42.00

**Table 10:** Availability of the blood transfusion policy and guidelines.

Variable	Frequency	Percentage (%)
Do you have a written policy for the administration of blood in your ward?		
Yes	77	28.73
No	137	51.12
Don't know	54	20.15
If yes, have you read the policy?		
Yes	71	92.21
No	6	7.79

**Table 11:** General knowledge of nurses on blood transfusion administration at KATH.

Variable	Frequency	Percentage		
		Mean	Min	Max
Total knowledge score	279	29	3	53
Respondents who scored more than 29%	129	46.24		
Respondents who scored less than 29%	150	53.76		

## Data Availability

The data obtained and used in this study are available from the corresponding author upon request.

## Additional Points

*Study Limitations*. As knowledge does not always translate into practice, an observational study on safe blood transfusion practices was necessary. However, on account of the limited time available for the study, an observational component could not be incorporated into the methodology.

## References

[1] World Health Organization (April 2020). *Blood Transfusion*.

[2] Armstrong B. (2008). Benefits and risks of transfusion. *ISBT Science Series*.

[3] Zaccheo M. M. (2009). *Implementation of an evidence-based bloodtransfusion education program in the intensive Cre unit: 5 evaluating nursing knowledge, Doctoral thesis, Chatham Univerity*.

[4] Tapko J. B., Mainuka P., Diarra-Nama A. J. (2009). *Status of Blood Safety in the WHO African Region: Report of the 2006 Survey*.

[5] Tetteh E. (2015). *Knowledge and Practices of Blood Transfusion Among Nurses in Ghana: Experiences from the Cape Coast Teahing Hospital, Cape Coast*.

[6] Bolton-maggs P. H. B., Cohen H. (2013). Serious Hazards of Transfusion (SHOT) haemovigilance and progress is improving transfusion safety. *British Journal of Haematology*.

[7] Ohsaka A. (2009). Transfusion errors and their prevention. *ISBT Science Series*.

[8] Sauer D. A., Mcdonald C. E., Boshkov L. (2001). Errors in transfusion medicine. *Laboratory Medicine*.

[9] Bradbury M., Cruickshank P., Jeremy (April 2018). Blood transfusion: crucial steps in maintaining safe practice. *The British Journal of Nursing*.

[10] Owusu-Ofori A. K., Owusu-Ofori S. P., Bates I. (2017). Detection of adverse events of transfusion in a teaching hospital in Ghana. *Transfusion Medicine*.

[11] Lwanga S. K., Tye C.-Y., Ayeni O. (1999). *Teaching Health Statistics: Lessons and Seminar Outlines*.

[12] Hijji B. M., Oweis A. E., Dabbour R. S. (2012). Measuring knowledge of blood Transfusion : a survey of Jordanian nurses. *American International Journal of Contemporary Research*.

[13] Kafando E., Koumare A. R., Sawadogo S., Nebie Y., Tinto A., Simpore J. (2017). Improving blood transfusion safety: a survey on the knowledge and attitudes of Health professionals in blood transfusion at the Yalgado Ouedraogo University hospital center, Burkina Faso. *Hematology, Transfusion and Cell Therapy*.

[14] Brunskill S., Thomas S., Whitmore E. (2012). What is the maximum time that a unit of red blood cells can be safely left out of controlled temperature storage?. *Transfusion Medicine Reviews*.

[15] WHO (2002). *The Clinical Use of Blood*.

[16] National Blood Service Ghana (2013). *National Guidelines for the Clinical Use of Blood and Blood Products in Ghana*.

[17] Khetan D., Katharia R., Pandey H. (2018). Assessment of bedside transfusion practices at a tertiary care center: a step closer to controlling the chaos. *Asian Journal of Transfusion Science*.

[18] Sapkota A., Poudel S., Sedhain A., Khatiwada N. (2018). Blood transfusion practice among healthcare personnel in Nepal: an observational study. *Journal of Blood Transfusion*.

[19] Elhy A. H. A., Kasemy Z. A. A. (2017). Nurses’ knowledge assessment regarding blood transfusion to ensure patient safety. *IOSR Journal of Nursing and Health Science*.

[20] Smith F. C., Donaldson J., Pirie L. (2010). Pre-registration adult nurses’ knowledge of safe transfusion practice: results of a 12 month follow-up study. *Nurse Education in Practice*.

[21] National Blood Service Ghana (2013). *Clinical Blood Transfusion Policy*.

[22] World Health Organization (WHO]) *Clinical Transfusion Practice: Guidelines for Medical Interns*.

[23] Saillour-Glennison F., Vialle S., Mathoulin-Pelissier S., Bouchon B. (2002). Factors associated with nurses’ poor knowledge and practice of transfusion safety procedures in Aquitaine, France. *International Journal for Quality in Health Care*.

[24] National Blood Users Group (2004). *Guidelines for the Administration of Blood and Blood Components*.

[25] Khalil S. S., Mohammad Z. A., El-deen M. E. E., Ahmed N. M. (2013). Impact of implementing a designed nursing intervention protocol on nurses’ knowledge and practice regarding patients undergoing blood transfusion. *The Medical Journal of Cairo University*.

[26] Asamani J. A., Amenorpe F. D., Babanawo F., Ofei A. M. A. (2014). Nursing documentation of inpatient care in eastern Ghana. *The British Journal of Nursing*.

[27] Buunaaisie C., Iddrisu O. A., China L. Y., Abass Y., Kyilleh J., Abdul-Malik A. (2018). *Project Report on Assessment of Nursing Documentation Practices in Five Hospitals in Tamale Metropolis: A Retrospective Records Review*.

[28] Kaur P., Kaur G., Kaur R., Sood T. (2014). Assessment of impact of training in improving knowledge of blood transfusion among clinicians. *Transfusion Medicine and Hemotherapy*.

[29] Lee E. L. S., Rahim N. A. A., Din S. A. T. (2016). Knowledge of blood transfusion among nurses at hospital pulau pinang: nursing responsibilities and patient management related to transfusion reactions. *Education in Medicine Journal*.

[30] Silva K. F. N. d., Duarte R. D., Floriano D. R. (2017). Blood transfusion in Intensive Care Units: knowledge of the nursing team. *Avances en Enfermería*.

[31] Yaghoobi M., Shirzae K., Sotvaan H., Yaghoobi E. P., Yoosefian N. M. (2014). Knowledge level of the staff about blood transfusions in hospitals in East of Iran. *Science Road Journal*.

[32] Erel M., Marcus E.-L., Dekeyser-ganz F. (2017). Barriers to palliative care for advanced dementia: a scoping review. *Annals of Palliative Medicine*.

[33] Tavares J. L., Barichello E., Mattia A. L. D., Barbosa M. H. (2015). Factors associated with knowledge of the nursing staff at a teaching hospital on blood transfusion. *Revista Latino-Americana de Enfermagem*.

[34] Mwamwenda T. S. (2014). Education level and human immunodeficiency virus (HIV)/acquired immune deficiency syndrome (AIDS) knowledge in Kenya. *Journal of AIDS and HIV Research*.

